# A Real-Time Path Planning Algorithm for AUV in Unknown Underwater Environment Based on Combining PSO and Waypoint Guidance

**DOI:** 10.3390/s19010020

**Published:** 2018-12-21

**Authors:** Zheping Yan, Jiyun Li, Yi Wu, Gengshi Zhang

**Affiliations:** Marine Assembly and Automatic Technology Institute, College of Automation, Harbin Engineering University, Harbin 150001, China; yanzheping@hrbeu.edu.cn (Z.Y.); ivy_wuyi@126.com (Y.W.); zgengshi@163.com (G.Z.)

**Keywords:** path planning, particle swarm optimization, waypoint guidance, autonomous underwater vehicle, forward looking sonar

## Abstract

It is a challengeable task to plan multi-objective optimization paths for autonomous underwater vehicles (AUVs) in an unknown environments, which involves reducing travel time, shortening path length, keeping navigation safety, and smoothing trajectory. To address the above challenges, a real-time path planning approach combining particle swarm optimization and waypoint guidance is proposed for AUV in unknown oceanic environments in this paper. In this algorithm, a multi-beam forward looking sonar (FLS) is utilized to detect obstacles and the output data of FLS are used to produce those obstacles’ outlines (polygons). Particle swarm optimization is used to search for appropriate temporary waypoints, in which the optimization parameters of path planning are taken into account. Subsequently, an optimal path is automatically generated under the guidance of the destination and these temporary waypoints. Finally, three algorithms, including artificial potential field and genic algorithm, are adopted in the simulation experiments. The simulation results show that the proposed algorithm can generate the optimal paths compared with the other two algorithms.

## 1. Introduction

Recently AUV technology has attracted much attention due to its wide use in commercial and military applications, such as ocean resources exploration and exploitation, hydrogeology survey, submarine cable inspection, mine detecting and sweeping, and harbor safety supervision [1,2,3,4]. 

In the past decades, a number of approaches have been applied in path planning for autonomous underwater vehicles, such as the Dijkstra algorithm [5], A* algorithm [6], polar histogram algorithm, grid method, artificial potential field algorithm (APF) [7], waypoint guidance method [8,9], fuzzy approach [10,11], genic algorithm (GA) [12], and particle swarm optimization (PSO) [13,14]. Path planning is divided into two classifications: global path planning and local path planning. Global path planning aims at searching for a feasible path in a known environment, and generally, the optimization path is available. Local path planning is adopted in unknown or partly unknown environments. In this situation, the optimization path is not always available. Two types of path planning have different emphases, the former demands these algorithms have global search ability to gain the optimal path, but it neither needs the algorithm running online nor limits the algorithm’s operating time. However, the latter is more focused on the algorithm’s capacity of rapid response to dynamic environments, and needs the algorithm running online and a fast response speed.

Graph search methods like A* algorithm, D* algorithm, and grid method have been employed for global path planning, as they are suitable for planning paths in known environments [15]. However, much of the ocean environment is still unknown, and the graph search algorithms are seldom used solely in AUV’s path planning in ocean environments [6]. Grid method is suitable for path planning in known environments too. Although it has the advantage in searching for the optimal path free of collision, the huge storage burden in vast range path planning and show response to dynamic obstacles limits its widely practical applications [16]. APF has advantages in its simple structure and easy implementation, but it is prone to getting stuck in the local minimum when AUVs run into clutter obstacle environments or corridor terrain, and the trajectory generated by this method is not smooth [7,17]. 

Genetic algorithm (GA) and particle swarm optimization (PSO) are typical evolutional algorithms. GA is a research algorithm stimulated by natural evolution, it incorporates survival of the fittest evolutionary including conventional evolutionary operators such as crossover and mutation. GA may converge to a suboptimal solution after dozens of generation evolution [18]. PSO is also a parallel random search algorithm, which selects the optimal value from the swarm as the evolutionary goal for the next iteration, and the search is continued until the iteration times expires, or error satisfies predefined desire [13,19]. 

Path planning technologies are progressing with existing algorithms being improved, and new algorithms being proposed. Hybrid approaches have been widely adopted in recent years, using multiple approaches’ merits to overcome the drawbacks of a sole approach [20,21,22]. Reference [11] presented a path planning method that combined adaptive fuzzy control and GA. Other classical methods such as the neural network merging fuzzy inference system was presented in Reference [23].

As an important research aspect of AUV technologies, path planning involves shortening of traveling distance, reducing travel time, smoothing trajectory, and keeping a safe distance to obstacles (safe margin). However, most of the above papers concerning path planning are based on obstacles of regular shape (e.g., rectangle, circle, sphere). In fact, obstacles’ shapes are irregular in most cases. In addition, those algorithms don’t take into account AUVs’ turning restraints, including turning radii and angle velocities, and therefore the planned plans are neither smooth, nor difficult for the AUV to track on. Several path smoothness technologies are proposed in these works [24,25,26].

To address aforementioned issues and design optimal paths for AUV in unknown complex environments, this paper presents a new path planning algorithm combined particle swarm optimization with waypoint guidance (PSO-WG). The concept of PSO-WG is as below: (1) Forward looking sonar (FLS) is adopted to detect the obstacles existing in the environment, these obstacles are transformed as polygons; (2) the search districts of PSO are elaborately devised to shorten the search time of PSO by getting rid of useless space; (3) PSO is utilized to search for temporary waypoints in the shrunken districts for obstacle avoidance, and an optimal path is generated under the guidance of temporary waypoints and destination. PSO-WG is a real-time algorithm, it can respond rapidly to dynamic environments. In addition, the obstacle avoidance trajectory can be planned by PSO in advance, so AUV keep a constant speed over grand (SOG) in the whole travel process. Furthermore, the path planned by this method is smooth in consideration of AUV turning peculiarity.

The main contributions of this paper are as follows: (1) The search range of PSO is reduced, which shortens the running time of the algorithm vastly and removes the possibility of producing infeasible paths; (2) the important parameters affecting the performance of planned path are adjustable in PSO algorithms, which makes it possible for this algorithm to be flexible enough to satisfy multiple task requirements; and (3) path planning is conducted by combining PSO and waypoint guidance, which simplifies the process of path planning and produces a smooth path for AUV as well.

The rest of the paper is organized as follows: Section 2 presents the AUV kinematics model description and formulation. Section 3 presents the hybrid path planning based on PSO algorithm and waypoint guidance. In Section 4, the simulation is conducted, and results are provided to illustrate the performance of the presented algorithm. Finally, conclusions are given in Section 5.

## 2. Problem Description and Formulation

### 2.1. Kinematics Model

In this paper, the AUV was equipped with a main propeller, horizontal rudder, and vertical rudder. The main propeller was mounted at the stern providing navigation power (surge), and the horizontal rudder and vertical rudder were utilized to change the heading angle of AUV in the vertical and horizontal direction, respectively. Therefore, surge, pitch, and yaw were controllable. 

Two reference coordinates were adapted in this paper, they are North-East-Down (NED) coordinate and body-fixed coordinate. A six free degrees kinematics model for AUV is described as follows [27]:

(1) $$\left[\begin{array}{c} \dot{x}\\ \dot{y}\\ \dot{z} \end{array}\right]=\left[\begin{array}{ccc} \cos\psi \cos\theta & \cos\psi \sin\theta \sin\phi -\sin\psi \cos\phi & \cos\psi \sin\theta \cos\phi +\sin\psi \sin\phi \\ \sin\psi \cos\theta & \sin\psi \sin\theta \sin\phi +\cos\psi \cos\phi & \sin\psi \sin\theta \cos\phi -\cos\psi \sin\phi \\ -\sin\theta & \cos\theta \sin\phi & \cos\theta \cos\phi \end{array}\right]\cdot \left[\begin{array}{c} u\\ v\\ w \end{array}\right]$$

(2) $$\left[\begin{array}{c} \dot{\phi }\\ \dot{\theta }\\ \dot{\psi } \end{array}\right]=\left[\begin{array}{ccc} 1 & \sin\phi \tan\theta & \cos\phi \tan\theta \\ 0 & \cos\phi & -\sin\phi \\ 0 & \sin\phi /\cos\theta & \cos\phi /\cos\theta \end{array}\right]\cdot \left[\begin{array}{c} p\\ q\\ r \end{array}\right]$$

**Hypothesis** **1:** *As the roll movement is uncontrollable for the AUV in this paper and the structure of AUV is bilateral symmetrical, let φ = 0, w = 0, v = 0, so formula (1), (2) can be rewritten as:*

(3) $$\left[\begin{array}{c} \dot{x}\\ \dot{y}\\ \dot{z} \end{array}\right]=\left[\begin{array}{l} \cos\psi \cos\theta \\ \sin\psi \cos\theta \\ -\sin\theta \end{array}\right]\cdot u$$

(4) $$\left\{ \begin{array}{l} \dot{\theta }=q\\ \dot{\psi }=r/\cos\theta \end{array}\right.$$

### 2.2. Sonar Model

A forward looking sonar (FLS) was installed onboard the AUV for obstacle detection. The major parameters of FLS are as follows: the detection range *L_e_* = 120 m, detection frequency *f* = 1 Hz, vertical detection angle is 3°, and horizontal detection width $\varphi_{s}$ is 120°. 

**Figure 1:**
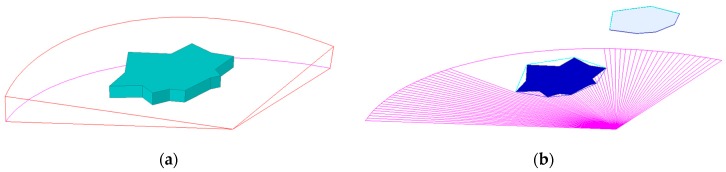
Obstacle shape transformation. (**a**) Obstacle’s real shape; **(b)** Obstacle is changed into polygon, where the blue lines represent the detectable outline.

In this paper, a real-time obstacle avoidance strategy was executed relying on FLS, all the obstacles are considered unknown and their shapes are irregular, and the obstacle outlines are generated according to the detection data of FLS. Some treatments have been made for obstacles before AUV detours around these obstacles. In planar obstacle avoidance, these obstacles are changed into convex polygons by the largest polar angle algorithm (LPAA) [28]. Figure 1 shows the result of obstacle shape transformation, where the obstacle (green object in Figure 1a) is transformed into the gray polygon in planar path planning. In fact, only part of obstacle outline is detected by FLS, the other part is shadowed by itself, the detectable part of the FLS view is represented in blue lines.

## 3. Hybrid Path Planning Algorithm Based on PSO and Waypoint Guidance

The PSO-WG algorithm proposed in this paper is a hybrid path planning algorithm combining particle swarm optimization and waypoint guidance algorithm. In this approach, the AUV heading was adjusted automatically under the guidance of the destination and temporary waypoints, the desired heading was always pointed to the destination if there was no obstacle in range of the FLS so those obstacles did not collide with the AUV. Otherwise, the PSO was utilized to generate a temporary waypoint in range of FLS, and the temporary waypoint replaced the destination as the current goal of AUV.

### 3.1. Particle Swarm Optimization

Particle swarm optimization (PSO) is an evolutionary algorithm inspired by natural behavior of a bird swarm. The parallel research is implemented by multiple particles, which can automatically adjust their search direction and velocity towards the best position, and this algorithm is able to obtain the optimal result with great potential [14]. It has the characteristics of a simple structure, fast convergence, and ability to adaptively adjust the parameters. The particle positions and search velocities are updated as follows:

(5) $$v_{ij}(t+1)=wv_{ij}(t+1)+c_{1}r_{1}(p_{ij}(t)-x_{ij}(t))+c_{2}r_{2}(p_{gj}(t)-x_{ij}(t))$$

(6) $$x_{ij}(t+1)=x_{ij}(t)+v_{ij}(t+1)$$

where *c*_1_, *c*_2_ (social component) are the acceleration coefficients, *r*_1_, *r*_2_ are random numbers uniformly distributed within the range, *w* is inertia weight of search speed, *i* is the *i*th particle, *j* is the *j*th dimension of particle, *t* is the iteration number, *p_ij_* (“personal best”) is the previous best position of the *i*th particle, and *p_gj_* (“global best”) is the previous best position among all the particles.

In general, a large value for *c*_1_, *c*_2_ led to faster convergence, which was beneficial for particles at an early stage of convergence, however, in the later period, large values for *c*_1_, *c*_2_ led to particles missing the best position and increased the probability of getting stuck in local optima. Therefore, the coefficients related to search were self-adjusted by the following equations:

(7) $$c_{1}=(c_{1\min}-c_{1\max})(i_{t}/i_{tol})+c_{1\max}$$

(8) $$c_{2}=(c_{2\max}-c_{2\min})(i_{t}/i_{tol})+c_{2\min}$$

(9) $$w=w_{\max}-(w_{\max}-w_{\min})(i_{t}/i_{tol})$$

where, *c*_1min_, *c*_1max_, *c*_2min_, *c*_2max_ are the lower limit and upper limit of acceleration coefficients, respectively, *w*_min_, *w*_max_ are the lower limit and upper limit of inertia weight, respectively, *i*_tol_ is the total iteration.

### 3.2. Hybrid Path Planning Algorithm

Generally, waypoint guidance path planning method is adopted in the known environment, in which the method can design a path rapidly. However, it is seldom solely utilized in unknown underwater environments with plenty of uncertain obstacles. As obstacles can be detected by FLS, the method can be adopted to avoid obstacles in the local range detected by the FLS. 

Currently, PSO is widely used in path planning, and it has the benefits of quick search and better reliability in finding the optimal path. However, some problems exist in PSO path planning in previous papers, which limits PSO being widely applied in path planning. Firstly, it is difficult to design PSO encoding in real-time path planning. Secondly, it is difficult to take vehicle turning constraints into account in the PSO algorithm, which led to those paths planned by this approach being absent of sufficient smoothness. Like other path planning algorithms, PSO cannot guarantee every planned path free of collision with obstacles. Therefore, some procedures are needed to validate that those paths are feasible, and the infeasible paths need to be revisited. 

**Figure 2:**
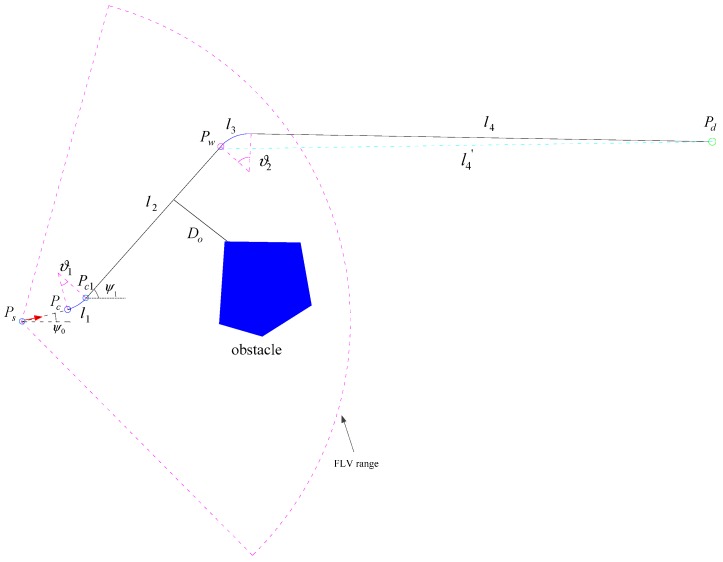
PSO-WG algorithm schematic.

This paper presents a new real-time path planning algorithm that is the PSO-WG algorithm, in which a temporary waypoint is generated in the range of FLS when an AUV needs to detour obstacles, and PSO is adopted to find the appropriate position of waypoint. Temporary waypoints and the destination are the goal position for an AUV to arrive, they guide the AUV’s heading and a suitable, smooth path is generated under this guidance. Temporary waypoints are plotted prior to the destination, temporarily replacing the destination as the goal position, and they are expired when the AUV arrives at them, the principle of PSO-WG algorithm is shown below in Figure 2. 

The schematic of the PSO-WG algorithm is shown in Figure 2, where *P_s_* (*x_s_*, *y_s_*) is the position for designing temporary waypoints. The AUV’s current heading ($\psi_{0}$) is denoted by the red arrow, *P_c_* (*x_c_*, *y_c_*) is the starting detouring obstacle position for AUV, *P_w_*(*x_wp_*, *y_wp_*) is the temporary waypoint, *P_d_* (*x_d_*, *y_d_*) is the destination, *P_c*1*_* (*x_c*1*_*, *y_c*1*_*) is the position of ending steering, and *D_o_* is the nearest distance between the AUV and obstacle in obstacle avoidance. The temporary waypoint is designed when an AUV will collide with obstacles or the safe margin will not be maintained if the AUV keeps the current heading angle. *P_s_* is the place for designing temporary waypoints where the distance among AUV and the nearest obstacle is about 60 meters, 1–2 s is set aside for the operation time of the algorithm, *P_c_* is the position of the AUV when it begins driving to the temporary waypoint with a constant turning radius *R_T_*. The temporary waypoint *P_w_* is expired when the AUV arrives at it, and the AUV turns to the destination again with the same turn radius. 

### 3.3. Planar Path Planning

In some special tasks, AUVs are required to travel in a predefined water depth. In this situation, planar path panning is needed. As shown in Figure 2, the new path consists of two parts, one is from the start of the detouring obstacle position *P_c_* (*x_c_*, *y_c_*) to the temporary waypoint *P_w_*(*x_wp_*, *y_wp_*), and the other is from the temporary waypoint to the destination *P_d_* (*x_d_*, *y_d_*). The first part of the path is precise, but the other one is a rough evaluation as the environment is unknown. In this algorithm, the planned path is smooth, it is generated under the guiding of the temporary waypoints and destination, and *arc l*_1_ and arc *l*_3_ are the turning trajectories.

Path planning of AUV involves multi-objects optimization. For this, several factors need to be considered, which include energy consumption, travel time, safety, and smoothness of trajectory. The length of the planned path nearly reflects the energy consumption and time expenditure, and energy consumption is an important factor which has to be taken into account for limited energy carrying AUV. Energy is consumed in the process of navigation, changing flight direction, and diving. Safety is also paramount, which is embodied in keeping a safe distance away from obstacles (safe margin). Sometimes it is necessary to consider the travel time in a certain task. The optimum of path planning is expressed in fitness function J:

(10) $$\min J=\lambda_{1}L_{1}+\lambda_{2}L_{2}+\lambda_{3}\Delta \vartheta +S_{pri}$$

where *L*_1_, *L*_2_ denotes the path length from the current position to the waypoint and from the waypoint to the destination, respectively, β denotes the angle variation in the temporary path planning, *S_pri_* is the safety price that is related to the nearest distance to obstacle, and $\lambda_{1}$, $\lambda_{2}$, $\lambda_{3}$ are constant coefficients. 

The paths are comprised of two lines and two arcs, and the path from the waypoint to the destination is displaced by the ${l_{4}}^{\prime }$ when it exceeds 120 m. The re-planned paths satisfy:

(11) $$L_{1}=l_{1}+l_{2}$$

(12) $$L_{2}=l_{3}+l_{4}$$

(13) $$\beta =\vartheta_{1}+\vartheta_{2}$$

(14) $$\vartheta_{2}=\mathrm{atan}(\frac{y_{d}-y_{wp}}{x_{d}-x_{wp}})-\psi_{1}$$

As the second section path is a rough evaluation of the remaining journey, $\vartheta_{2}$ is approximately estimated by Equation (14). The waypoint and $\vartheta_{1}$ satisfies the following equations:

(15) $$l=R_{T}\vartheta_{1}$$

(16) $$x_{c1}=x_{c}+l\cos(\psi_{0}+0.5\vartheta_{1})$$

(17) $$y_{c1}=y_{c}+l\sin(\psi_{0}+0.5\vartheta_{1})$$

(18) $$\psi_{1}=\mathrm{atan}(\frac{y_{wp}-y_{c1}}{x_{wp}-x_{c1}})$$

(19) $$\vartheta_{1}=\psi_{1}-\psi_{0}$$

(20) $$S_{Pri}=\{\begin{array}{c} 0,\;D_{o}\geq 2L_{o}\\ \lambda_{4}{\left(2L_{o}/D_{o}\right)}^{2}\\ \infty ,D_{o}<0 \end{array},else$$

where $\psi_{0}$ is AUV’s current heading, $\psi_{1}$ is the heading of AUV when AUV finishes turning to the temporary waypoint. Combined with Equations (5)–(19), the optimal value of the fitness function *J* can be obtained, which is related to the waypoint. 

In general, there exist the following problems in path planning: (1) Large search ranges lead to plenty of time being consumed in search of the optimal path; (2) safe margins are not enough or the planned path runs across an obstacle, which often occurs, then some measures need to be taken for verifying and correcting the problems; and (3) the designed path is not smooth or does not consider the turn restriction of AUV. 

In order to solve the above problems, some improvements have been presented in this paper. Firstly, in the PSO-WG algorithm, the turn restriction of AUV is taken into account, which solves the problem that the planned path is not smooth. Secondly, in this approach, we devise the fitness function in PSO algorithm, where safe margin, path length, and time consumption are comprehensive considered, and the performance of the planned path can be adjusted according to the required tasks. Moreover, the operation time of PSO-WG in finding temporary waypoints is largely shortened via limiting the search range of PSO. 

**Figure 3:**
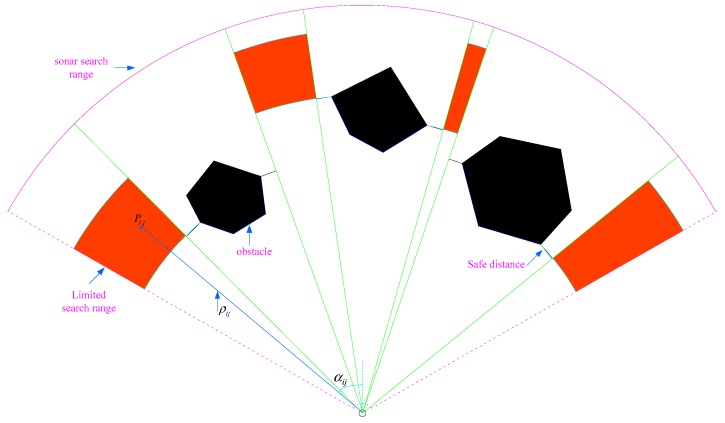
Shrunken search range of PSO in planar.

The shrunken search range of PSO is shown in Figure 3, where the detection range of sonar is the regions enclosed by purple lines, the black blocks are obstacles, and the decreased search ranges of PSO are the red blocks. In designing the search ranges of PSO, we have taken into account the safe distance to obstacles. Waypoints are only produced in the red region, and therefore, the trajectories from the AUV current position to waypoints are safe and feasible. In addition, the search ranges of PSO cannot be too close to the external edge of the sonar detection range, in case the obstacle is so close that it is difficult to plan a safe path for AUV. It is best to keep about a 30 m margin between the limited search range upper border and sonar external border if possible. 

The two-dimensional PSO-WP path planning procedure is as follows: Particles are distributed to different districts with the same distribution density, which means the number of particles in each district is variable with the district size. A particle’s position is denoted arbitrarily by polar coordinates as [$\rho_{ij},\alpha_{ij}$], where $\rho_{ij}$ denotes its distance to sonar and $\alpha_{ij}$ denotes a particle’s polar angle, the coordinate origin is taken as the sonar position, and the polar axis is taken as the center line of sonar range. Particle position and particle search velocity are both two-dimension scalar. The coordinates of a waypoint can easily be transformed from polar coordinate style to NED coordinate as follows:

(21) $$x_{wp}=x_{c}+\rho_{ij}\cos(\alpha_{ij})$$

(22) $$y_{wp}=y_{c}+\rho_{ij}\sin(\alpha_{ij})$$

where *x_c_*, *y_c_* are the coordinates of sonar. Those particles are equally dispersed in the search range with the same density initially. The positions and search velocities of particles need to be updated at the beginning of the next search according to Equations (5)–(9), those parameters need rectification whenever they exceed their upper or lower limits.

**Figure 4:**
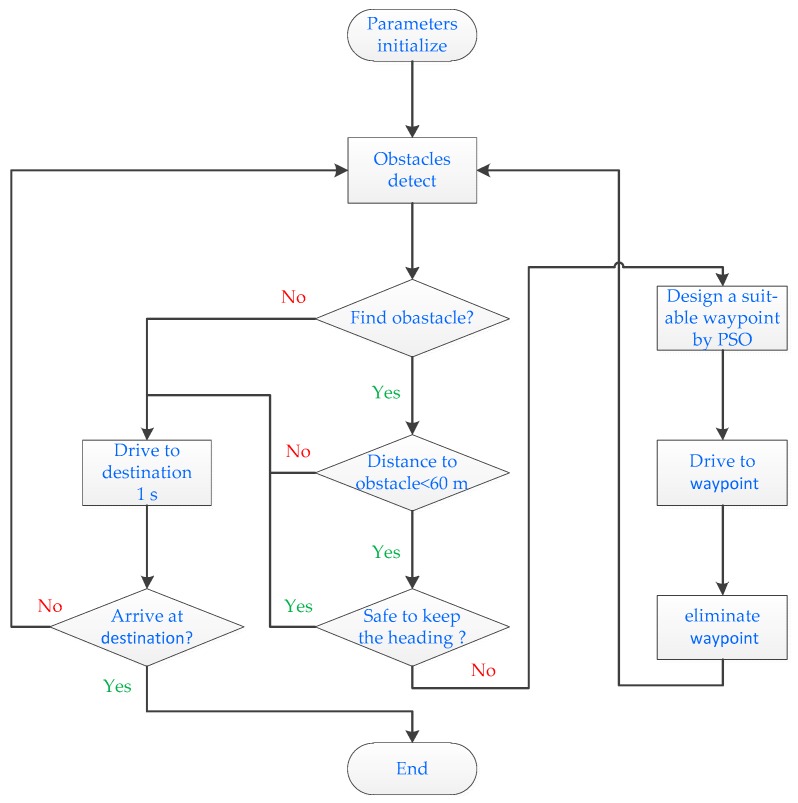
The flow chart of path planning.

As the search range is divided into several separate districts, each district needs an individual search to find a candidate waypoint. The position of the particle whose fitness function value is the smallest is selected as the candidate waypoint. The optimal waypoint is selected from above candidate waypoints as the temporary waypoint. The flow chart of path planning is shown in Figure 4.

## 4. Simulation Results

In order to verify the effectiveness of the proposed path planning algorithm in an unknown environment, three algorithms including APF and GA algorithms are adopted in the path planning simulations. The simulations are conducted in MATLAB 2015a, and the PC is configured with Intel core is-4005U at 1.7 GHz, 4G RAM. 

### 4.1. Static Environment Path Planning

The simulation environment was designed as follows: The size of the scene was 500 × 600 m^2^ and 14 irregular obstacles were distributed in the scene. The AUV initial position was (20, 20), the heading angle was 0° (north), SOG was 2 m/s, the destination was (450, 540), and the simulation step size was 0.1 s. The FLS parameter was the same as those in the previous FLS model. All the obstacles in these scenes were irregular polygons and their positions were unknown in advance, they were discovered when they entered the detection range of FLS. The parameters for the PSO algorithm were selected as: The distribution density of particles was one particle per 10 m^2^, the maximum number of iterations was 50, terminated tolerance was 0.1, inertia weight *w* linearly decreased from 0.9 to 0.4, learning coefficient *c*_1_ linearly decreased from 2.5 to 0.5, and *c*_2_ linearly increased from 0.5 to 2.5. The other parameters were designed as follows:

$$L_{o}=\;4\;m,\;R_{T}=\;20\;m,\lambda_{1}=\lambda_{1}=1.0,\;\lambda_{3}=0.2R_{T},\;\lambda_{4}=5.0$$

**Figure 5:**
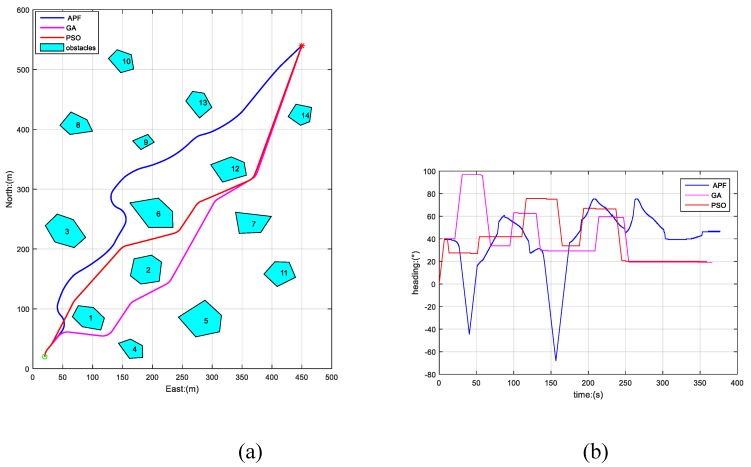
Paths planned by APF, GA, and PSO-WG in static environment. (**a**) The trajectories generated by APF, GA, and PSO-WG; (**b**) heading curves of AUV.

Figure 5 depicts the paths planned by APF, GA, and PSO-WG. In this scene, green “o” is the initial position of AUV, and red “*” is the destination, the blue curve is the path planned by APF, the magenta curve is the path planned by GA, and the red curve is the path planned by PSO-WG. It is easy to observe that the three paths are safe where the AUV keeps a safe distance to obstacles (safe margin) from Figure 5a. In Figure 5b, the time consumptions of AUV traveling are observable, and the smoothness of path are well expressed by the heading curve. Smoothness is embodied in the heading variation range and rate too, and the smoothest path was produced by PSO-WG, while the least smooth was produced by APF. 

**Figure 6:**
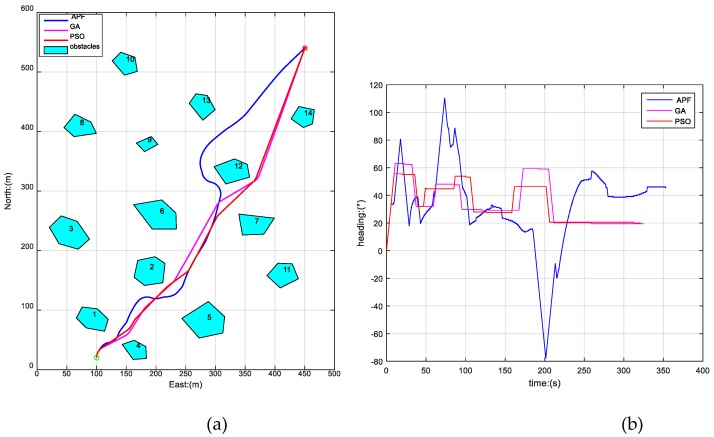
Paths planned by APF, GA, and PSO-WG in another initial position. (**a**) The trajectories generated by APF, GA, and PSO-WG; (**b**) heading curves of AUV.

**Figure 7:**
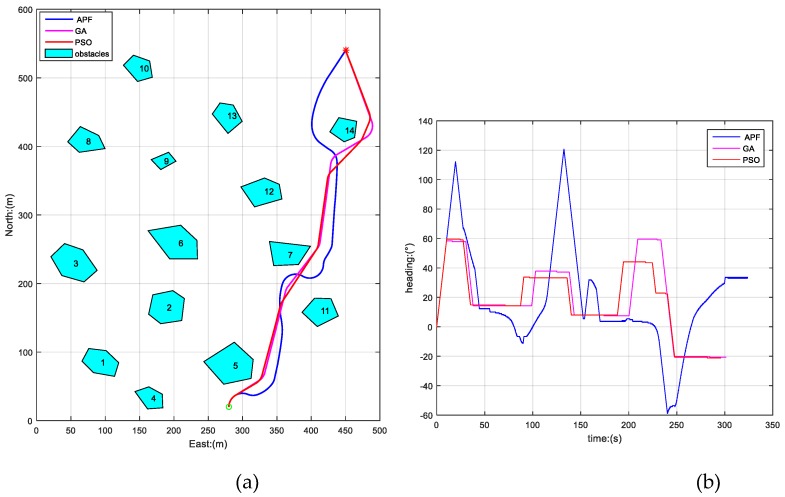
Paths planned by APF, GA, and PSO-WG in the third initial position. (**a**) The trajectories generated by APF, GA, and PSO-WG; (**b**) heading curves of AUV.

To further verify the above conclusion, we also carried out another two simulations by changing the initial position of AUV, while the other parameters were kept the same as before, the results of which are shown in Figure 6 and Figure 7. The path lengths and time consumptions of the three algorithms in Figure 5, Figure 6 and Figure 7 are shown in Table 1.

From Figure 5, Figure 6 and Figure 7 and Table 1, we can draw the conclusion that the three algorithms have all produced safe paths for AUV from different initial positions to the destination in a static unknown environment. The PSO-WG algorithm has planned the optimal path with shortest length, the least travel time, and the best smoothness, and the AFP algorithm has planned the worst path of the three algorithms.

### 4.2. Dynamic Environment Path Planning

**Figure 8:**
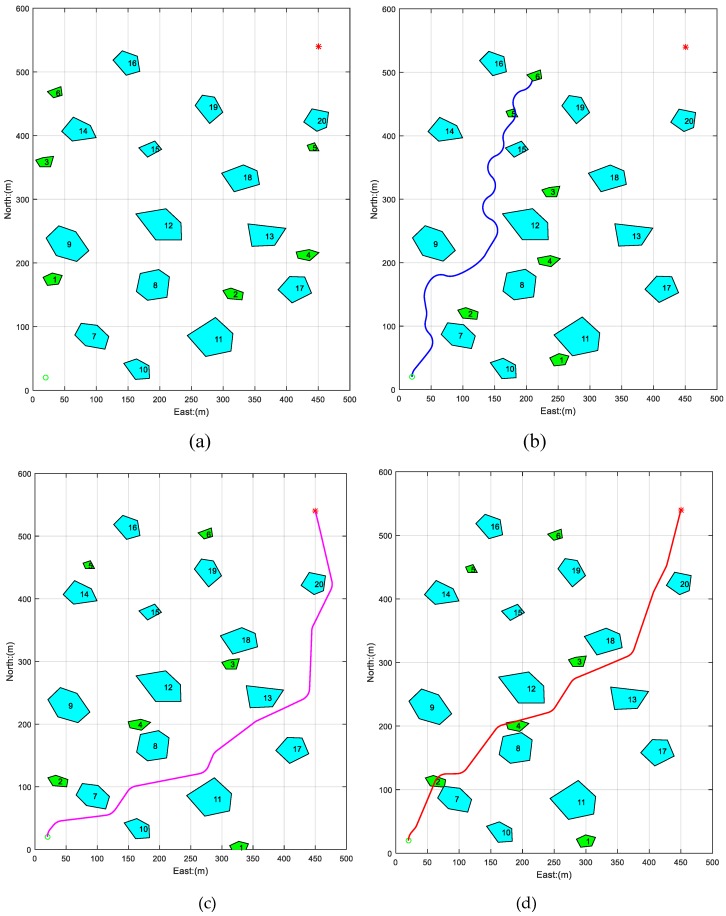
Path planned by APF, GA, and PSO-WG in a dynamic environment. (**a**) The initial distribution of obstacles in a dynamic scene; (**b**) the path planned by APF; (**c**) the path planned by GA; and (**d**) the path planned by PSO-WG.

In order to verify this method’s adaption in a complicated dynamic environment, six irregular moving obstacles were added in the above scene, and the result is shown in Figure 8.

In Figure 8a, the static obstacle’s distribution is the same as those in the static scene, and the moving obstacles are represented by green polygons. In Figure 8b, the path was planned by APF. AUV successfully avoided moving obstacles one and five, however, when the running time was about 299.8 s, the AUV encountered three obstacles including moving obstacle six, obstacle 16, and obstacle 19, where the APF was trapped in local minimum and the AUV collided with moving obstacle six.

In Figure 8c, the path was planned by GA. The path’s length was 809.2 m, an increase of about 56 m compared with the previous path in the static scene. In Figure 8d, the path was planned by PSO-WG. The length of the path was 750.6 m, an increase of about 18 meters compared with the previous path in the static scene.

**Figure 9:**
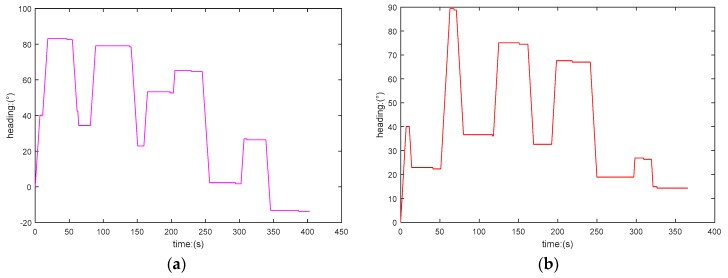
Heading angles of AUV. (**a**) Heading curve of AUV in GA; (**b**) heading curve of AUV in PSO-WG.

In Figure 9, the time consumption for the AUV traveling the paths planned by GA and PSO-WG is 404.6 s and 375.3 s, respectively. Comparing Figure 9a with Figure 9b, we can observe that the heading variation range of the former is bigger than the latter, that is to say the smoothness of the path panned by PSO-WG is more optimal. We thus conclude that the PSO-WG algorithm plans the optimal paths in dynamic environments too. 

From above results, it is easy to understand that the PSO-WG algorithm is the most beneficial for path planning in both static and dynamic environments in the following aspects: The trajectory is smooth, the length of the planned path is shorter (shorter travel time), and the AUV’s heading is more stable. 

## 5. Conclusions and Future Work

In this paper, an algorithm for combining particle swarm optimization and waypoint guidance has been proposed to plan an optimal path for autonomous underwater vehicles in unknown dynamic environments. In the process of path planning, several important aspects such as path length, time of traveling, safe margin, and smoothness of path are taken into account. Firstly, collision avoidance is conducted by the guiding of temporary waypoint. It is easy to adjust the parameters of fitness function to generate suitable paths according to the requirements of the tasks. Secondly, collisions with obstacles are pre-judged, and obstacle avoidance is planned in advance, so that autonomous underwater vehicles keep constant speed over ground for the entire navigation process. In addition, the turning constraints of autonomous underwater vehicles are taken into account, so the planned path is smooth and easy to be tracked for autonomous underwater vehicles.

Finally, simulations were implemented in two scenes with three different methods. The results validate that the proposed algorithm was able to plan a feasible path in a complicated environment, and its performance is the best compared with the other two algorithms.

The next stage of this work is to carry out this algorithm in a pool experiment, where we will extend this algorithm to more complicated three-dimensional environments with clutter dynamic objects.

## Figures and Tables

**Table 1:** The results of path planning in a static environment.

	APF		GA		PSO	
	P.L.: m	T.C.: s	P.L.: m	T.C.: s	P.L.: m	T.C.: s
1	753.4	376.7	706.2	353.1	647.8	323.9
2	732.8	366.4	649.8	301.5	603.0	301.5
3	718.4	359.2	642.8	321.4	592.0	296.0

Where P.L. is the abbreviation of path length, and T.C. is the abbreviation of time consumption.
